# Prevalence of Depression and Posttraumatic Stress Disorder in Flint, Michigan, 5 Years After the Onset of the Water Crisis

**DOI:** 10.1001/jamanetworkopen.2022.32556

**Published:** 2022-09-20

**Authors:** Aaron Reuben, Angela Moreland, Salma M. Abdalla, Gregory H. Cohen, Matthew J. Friedman, Sandro Galea, Alex O. Rothbaum, Michael G. Schmidt, John E. Vena, Dean G. Kilpatrick

**Affiliations:** 1 Department of Psychology & Neuroscience, Duke University, Durham, North Carolina; 2 Department of Psychiatry and Behavioral Sciences, Medical University of South Carolina, Charleston; 3 School of Public Health, Boston University, Boston, Massachusetts; 4 Department of Epidemiology, Boston University, Boston, Massachusetts; 5 Department of Psychiatry, Geisel School of Medicine at Dartmouth, Hanover, New Hampshire; 6 National Center for PTSD, US Department of Veterans Affairs, Washington, DC; 7 Department of Microbiology and Immunology, Medical University of South Carolina, Charleston; 8 Department of Public Health Sciences, Medical University of South Carolina, Charleston

## Abstract

**Importance:**

Environmental disasters, such as the Flint, Michigan, water crisis, are potentially traumatic events (PTEs) that may precipitate long-term psychiatric disorders. The water crisis was associated with acute elevations in mental health problems in the Flint community, but long-term psychiatric sequelae have not yet been evaluated using standardized diagnostic measures.

**Objective:**

To investigate the prevalence of and factors associated with current presumptive diagnostic-level major depression and posttraumatic stress disorder (PTSD) among Flint residents 5 years after the onset of the crisis.

**Design, Setting, and Participants:**

In this cross-sectional study, a household probability sample of 1970 adults living in Flint, Michigan, during the crisis were surveyed about their crisis experiences, their psychological symptoms 5 years later, and their access to and use of mental health services in the intervening years. Analyses were weighted to produce population-representative estimates.

**Main Outcomes and Measures:**

Presumptive *Diagnostic and Statistical Manual of Mental Disorders* (Fifth Edition) (*DSM-5*) diagnostic-level past-year major depression and PTSD.

**Results:**

Among 1970 respondents, 1061 of 1946 reporting sex (54.5%) were women; 1043 of 1951 reporting race (53.5%) were Black or African American and 829 (42.5%) were White; and 1895 of 1946 reporting ethnicity (97.4%) were non-Hispanic. Overall, 435 (22.1%) met *DSM-5* criteria for presumptive past-year depression, 480 (24.4%) for presumptive past-year PTSD, and 276 (14.0%) for both disorders. Residents who believed that their or their family’s health was harmed by contaminated water (eg, risk ratio [RR] for depression: 2.23; 95% CI, 1.80-2.76), who had low confidence in public-official information (eg, RR for PTSD, 1.44; 95% CI, 1.16-1.78), who had previous exposure to PTEs (eg, RR for both disorders: 5.06; 95% CI, 2.99-8.58), or who reported low social support (eg, RR for PTSD, 2.58; 95% CI, 1.94-3.43) had significantly higher risk for depression, PTSD, and comorbidity. PTEs involving prior physical or sexual assault were especially potent risk factors (eg, both disorders: RR, 7.30; 95% CI, 4.30-12.42). Only 685 respondents (34.8%) were ever offered mental health services to assist with water-crisis–related psychiatric symptoms; most (543 [79.3%]) who were offered services utilized them.

**Conclusions and Relevance:**

In this cross-sectional study of psychiatric disorder in Flint, Michigan, presumptive depression and PTSD were highly prevalent 5 years after the onset of the water crisis. These findings suggest that public-works environmental disasters have large-scale, long-term psychological sequelae. The Flint community may require expanded mental health services to meet continued psychiatric need. National disaster preparedness and response programs should consider psychiatric outcomes.

## Key Points

**Question:**

What are the long-term psychiatric outcomes of environmental disasters, such as the Flint water crisis?

**Findings:**

In this cross-sectional household probability sample survey of 1970 adults living in Flint, Michigan, during the water crisis, more than one-fifth met criteria for presumptive past-year depression, nearly one-quarter for past-year presumptive posttraumatic stress disorder, and more than one-tenth for both disorders 5 years after the onset of the water crisis. Only 34.8% were ever offered mental health services to assist with water-crisis–related psychiatric symptoms; most (79.3%) who were offered services utilized them.

**Meaning:**

These findings suggest that public-works environmental disasters such as the Flint water crisis have lasting psychological sequelae and may require expanded mental health services to meet long-term psychiatric need.

## Teaser

This cross-sectional study investigates the prevalence of and factors associated with current presumptive diagnostic-level major depression and posttraumatic stress disorder (PTSD) among residents of Flint, Michigan, 5 years after the onset of the water crisis.

## Introduction

The Flint water crisis began on April 25, 2014, when Flint, Michigan, switched its water supply from Lake Huron and the Detroit River to the more corrosive Flint River and failed to properly treat the supply with anticorrosives to prevent lead and iron leaching from the system’s old water pipes.^1^ Authorities initially reassured residents that the water was safe and delayed issuing do-not-drink orders until September 25, 2015, after nongovernmental researchers documented high blood-lead levels among Flint children.^2^ In October 2015, the water supply was returned to Lake Huron, but water-lead levels did not officially test below federal limits until January 24, 2017.^3^ Consequently, many residents of Flint, a predominantly low-income and African American community, had extended exposure to drinking water with unsafe levels of bacteria, disinfection byproducts, and lead, a neurotoxicant.

This crisis, with its rapid onset and long duration, potential for personal and family member exposure to toxic substances, and misinformation from trusted officials, represents a potentially traumatic event (PTE) capable of precipitating or exacerbating psychiatric disorders, particularly depression and posttraumatic stress disorder (PTSD), that may have long-term consequences for community mental health. (The *Diagnostic and Statistical Manual of Mental Disorders* [Fifth Edition] [*DSM-5*] defines a PTE as direct or indirect exposure to actual or threatened death, serious injury, or sexual violence; full definition appears in eAppendix 1 in the Supplement.)^4^ Lead exposure, which impairs central nervous system processes, also increases risk for behavioral and mental health problems via alterations in mood, personality, and diverse psychiatric symptoms.^5,6,7,8^ Finally, uncertainty about the extent of exposure to toxic substances and concerns about long-term health consequences may increase the risk of stress-related behavioral and mental health problems, particularly depression and PTSD.^9,10^

Early surveys of Flint residents during and shortly after the water crisis onset identified elevations in PTSD symptoms among those who believed that they were exposed to poor-quality tap water^11^ and more diffuse concerns involving stress, anxiety, and depressive symptoms among residents worried about the health and economic consequences of the crisis (eg, impacts to fertility, decreased property values).^12,13^ Two small but representative surveys conducted in 2018 and 2019 suggested that mental health problems remained elevated, with high rates of probable PTSD symptoms (29.0%) and depression and anxiety disorder (between 13.1% and 26.3%) identified through Flint residents’ reports on brief (2- to 5-item) screening questionnaires.^14,15^ To our knowledge, no study to date has provided estimates of the long-term prevalence of depression or PTSD at the full *DSM-5* diagnostic level using standardized interviews.

This article reports survey results from a household probability sample of adults in Flint, Michigan, conducted 5 years after the onset of the water crisis. We assessed experiences related to the crisis (eg, initial emotional responses, beliefs about exposure and potential harm), prevalence of presumptive depression and PTSD (disorders that are most often elevated following large-scale disasters),^16^ and information about mental health service access and use. We also evaluated potential factors associated with persistent mental disorder, including sociodemographic characteristics, such as race and income; beliefs and experiences related to the water crisis, such as trust in announcements from public officials; and non–crisis-related risk factors, including prior exposure to PTEs, particularly those that involve physical or sexual assault^17^; and low social support.^10^

## Methods

### Data Collection and Sample

Data were collected from August 13, 2019, through April 10, 2020, via mail and web-based survey of an area probability household sample of adults (aged ≥18 years) from the Flint community identified using address-based sampling. Letters with a brief description of the study were sent to randomly selected households within a specified geographical area. One adult from each household was randomly selected for participation using the most-recent-birthday method and asked to complete a survey about their experiences with and responses to the water crisis via web or mail. Most data (98%) were collected before the COVID-19 pandemic (ie, before the March 11, 2020, World Health Organization pandemic declaration and before the March 10, 2020, confirmation of Michigan’s first COVID-19 cases). All sampling, participant recruitment, data collection, and weighting was conducted by Abt Associates, a national survey research firm. Participants gave written informed consent, and the study protocol was approved by the Institutional Review Board for Human Research at the Medical University of South Carolina. This study followed the Strengthening the Reporting of Observational Studies in Epidemiology (STROBE) reporting guideline for cross-sectional studies. Further details on sampling, data collection, and key measures are provided in eAppendix 2 in the Supplement.

Several procedures encouraged participation and willingness to provide candid responses about sensitive matters. Names and contact information were not attached to survey responses. Respondents were informed that we had a privacy certificate from the Department of Justice that provides total response confidentiality, including protection from disclosure via subpoena in federal or state court. Respondents who completed the survey were reimbursed $35 for their time.

### Survey Content

Respondents were asked questions via a highly structured, self-administered survey interview using branching-format questions. Surveys were available in English and Spanish and ascertained demographic characteristics, experiences and beliefs related to the water crisis, past and current mental health concerns, and preexisting risk factors.

#### Experiences and Beliefs Related to the Water Crisis

Respondents were asked whether they lived in homes directly affected by tap-water quality (yes or no), experienced physical health problems due to exposures (not at all, a little, a moderate amount, or a great deal) or knew someone who had, or experienced mental or emotional problems related to concerns about tap-water quality (yes or no). They were also asked about strategies used to limit exposure, cumulative out-of-pocket costs to implement strategies, and their confidence in information from public officials concerning the safety of their water, both during the crisis and at the time of assessment, 5 years later.

#### Presumptive Depression and PTSD

Depression was measured using the *DSM-5* criteria for major depressive episode (MDE) via a modified version of the National Women’s Study Depression Module previously used in National Institutes of Health– and National Institute of Justice–funded epidemiological surveys of depression prevalence following criminal victimization,^17^ terrorist attacks,^18^ and natural disasters.^19^ All 9 MDE symptoms were assessed. Based on recency of symptoms, we determined whether criteria were met during the past month, past year, or since the water crisis onset. Criteria were met if respondents reported depressed mood most of the day nearly every day or markedly diminished interest or pleasure in all or most activities most of the day nearly every day, with a total of 5 or more depression symptoms (eg, disrupted appetite, sleep, concentration). This measure demonstrated good reliability (eg, past-month symptoms: α = .83).

PTSD was measured using the National Stressful Events Survey (NSES) PTSD Module developed in conjunction with the *DSM-5* PTSD Workgroup.^20^ This module assesses exposure to any of 11 types of *DSM-5* PTEs (eAppendix 1 and eTable 1 in the Supplement), including combat exposure, serious accidents, life-threatening illnesses, or physical or sexual assault, and all 20 *DSM-5* PTSD symptoms as well as whether symptoms have resulted in significant distress or interfered with work, school, health, or other social functioning. It also determines how recently diagnostic criteria have been met (ie, any time during their life, within the past year, or past month). This module demonstrated excellent reliability (eg, past-month symptoms: α = .93).

#### Preexisting Risk Factors

In addition to assessment of prior PTE exposure (detailed previously), respondents were asked about social support via a modified 5-item version of the Medical Outcomes Study module that assesses social support over the past 6 months^21^ (eAppendix 1 and eTable 2 in the Supplement). Respondents were asked how often they had someone available to “help you if you were confined to bed,” “give good advice about a crisis,” “get together with for relaxation,” “confide in or talk with about your problems,” and “love you and make you feel wanted.” Response options ranged from none of the time (score, 1) to all of the time (score, 4) (scale range, 5-20). Based on results from prior studies of the September 11 terrorist attacks^18^ and hurricanes in Florida,^22^ we defined low social support as a score of 15 or less. This scale demonstrated good reliability (α = .86).

#### Mental Health Service Use

Respondents were asked whether “mental health services for stress or other problems were ever offered to you or your family to assist with concerns or problems associated with the Flint Water Crisis?” If they answered yes, they were asked whether they utilized the services.

### Statistical Analysis

Analyses followed 3 stages. First, we calculated the prevalence of negative experiences and beliefs related to the water crisis. Second, we calculated the prevalence of presumptive major depression and PTSD. Secondary follow-up tests then investigated potential factors associated with mental disorder using Poisson regression models with robust error variance,^23^ regressing the mental health outcomes separately onto 3 categories of personal characteristics and risk factors one at a time: (1) sociodemographic characteristics (race, sex, and income); (2) psychological risk factors related to the water crisis (concerns about health problems following contaminated-water exposure and low confidence in public-official information); and (3) psychological risk factors unrelated to the water crisis (past PTE exposure and low social support). Associations of psychological risk factors with the mental health outcomes were adjusted for the sociodemographic factors of race, sex, and income. Conservative sensitivity tests regressing the mental health outcomes on all sociodemographic characteristics and risk factors simultaneously were also run to identify which factors were significantly associated with mental health disorders when controlling for all the other factors in the model.

Third, we calculated the prevalence of mental health services being offered to and ultimately utilized by respondents. Secondary follow-up tests using Poisson regression models with robust error variance then investigated the association of (1) sociodemographic factors of race, sex, and income with the outcome of being offered mental health services and (2) mental health service use with past-year mental disorder.

All analyses were weighted to adjust for potential nonresponse bias due to nonparticipation and to produce population-representative estimates by first weighting to adjust for household size and likelihood of household nonresponse and then using iterative proportional fitting to align the characteristics of the sample to match Flint population benchmarks on sex, education, race and ethnicity, marital status, and household size using Census Bureau 2018 American Community Survey 1-year estimates for Flint, Michigan. Analyses were conducted in STATA version 17.0 (StataCorp) using the svyset command for weighting. Significance tests were 2-tailed, with α = .05.

## Results

A total of 10 000 addresses were sampled and mailed recruitment letters. There was no response to 6693 letters. There was no way to determine how many households had eligible adults, how many adults opened and read the letter, or how many passively refused. Another 1112 letters were undeliverable or sent to vacant addresses. The remaining 2195 households accessed the survey, reviewed the consent materials, and completed the eligibility-screening questions. Of these, 1970 (89.7%; 1061 of 1946 reporting sex [54.5%] women) completed the survey. There were 85 (3.9%) partial completions, 95 (4.3%) refusals, and 45 (2.1%) screen-outs. After accounting for estimates of household ineligibility to respond (eg, vacant addresses, addresses with residents only younger than 18 years), the overall estimated response rate was 28.4%. Overall, 1235 participants (63%) completed the survey online and 735 (37%) on paper. The final weighted sample was demographically representative of the adult Flint population (Table 1). Most self-identified as Black or African American (1043 of 1951 reporting race [53.5%]) or White (829 [42.5%]), and non-Hispanic (1895 of 1946 reporting ethnicity [97.4%]). Most (1043 of 1836 reporting income [56.8%]) reported earning less than $25 000 a year.

**Table 1.** Weighted Demographic Characteristics of Participants^a^

Variable	No. (%)
Sex	
No.	1946
Female	1061 (54.5)
Male	885 (45.5)
Education	
No.	1965
Less than high school diploma	295 (15.0)
High school diploma/GED	760 (38.7)
Some college/associate degree	654 (33.3)
4-y college graduate	163 (8.3)
Post graduate training	92 (4.7)
Race	
No.	1951
Asian	2 (0.1)
Black or African American	1043 (53.5)
Native American	5 (0.3)
>1 Race	41 (2.1)
Other	31 (1.6)
White	829 (42.5)
Hispanic ethnicity	
No.	1946
Hispanic	51 (2.6)
Non-Hispanic	1895 (97.4)
Marital status	
No.	1950
Married	552 (28.3)
Divorced or separated	398 (20.4)
Widowed	136 (7.0)
Never married	864 (44.3)
Household size, persons	
No.	1899
1	460 (24.2)
2	682 (35.9)
3	347 (18.3)
≥4	410 (21.6)
Annual income, $	
No.	1836
<25 000	1043 (56.8)
25 000-49 999	499 (27.2)
50 000-74 999	178 (9.7)
75 000-99 999	55 (3.0)
≥100 000	61 (3.3)

^a^ Demographic characteristics are weighted to match US Census Bureau 2018 American Community Survey 1-year estimates for demographics of adults in Flint, Michigan. The full sample included 1970 participants.

### Perceived Harm From the Water Crisis

**Figure 1.**
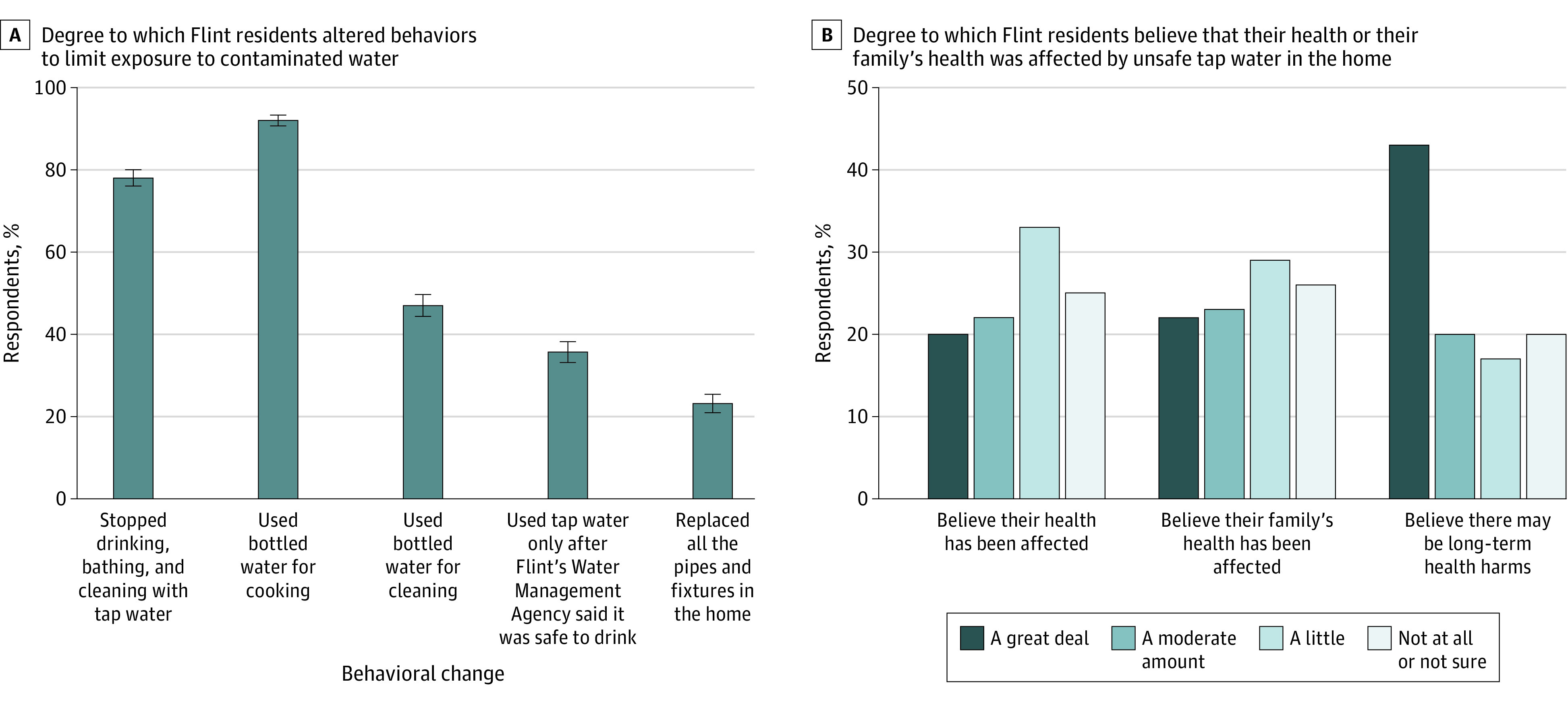
Altered Behaviors to Avoid or Reduce Exposure to Contaminated Tap Water and Beliefs That One’s Health or One’s Family’s Health Was Affected by Exposure Error bars represent 95% CIs.

Most respondents (86.8%) lived in homes directly affected by problems with tap-water quality during the crisis, and nearly all (97.7%) altered their behavior to avoid or reduce exposure to contaminated water, including avoiding drinking (78.0%), cooking (91.9%), or cleaning with (47.0%) tap water (Figure 1A). Most (76.9%) had to pay out of pocket to lower their risk, some in excess of $1000 (31.2%) and a few in excess of $10 000 (3.7%).

Despite efforts to limit exposure, most respondents believed their own health (75.3%) or their family’s health (73.8%) was affected in some way by exposure to contaminated water (Figure 1B). Most respondents were concerned that there will be long-term health effects (80.1%).

### Mental Health Concerns

Most respondents first learned about the water crisis from a news source (69.5%) or family member, friend, or acquaintance (10.9%). Nearly all (97.8%) experienced an immediate negative emotional response, ranging from concern and sadness to fear and anger. Five years later, 41.0% reported having experienced mental or emotional problems related to their concerns about water contamination.

**Figure 2.**
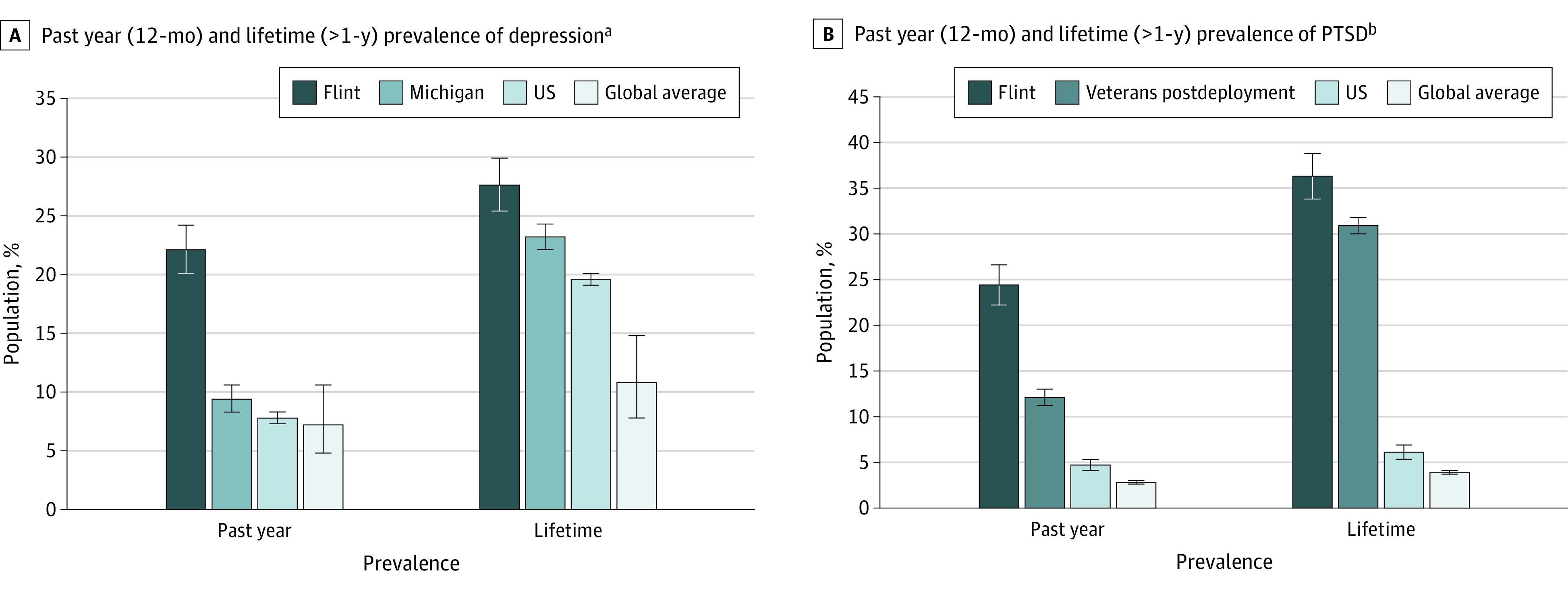
Prevalence of Mental Disorders Among Flint Residents Compared to Benchmark Data From Other Populations Error bars represent 95% confidence intervals. ^a^Past-year depression prevalence rates for contextual comparison are from the Centers for Disease Control Behavioral Risk Factor Surveillance Data for Michigan in 2010 (the last year 12-month prevalence was assessed), the 2019 National Survey on Drug Use and Health for the United States in 2019,^24^ and Lim et al^25^ for the global average from 1994 to 2014. Lifetime (>1 year) depression prevalence rates are from the Centers for Disease Control Behavioral Risk Factor Surveillance Data for Michigan in 2019 and the United States in 2018 and Lim et al^25^ for the global average. The National Survey on Drug Use and Health does not report standard errors for depression statistics, therefore for the purposes of constructing 95% CIs these have been assumed to match those reported for other mental disorders in the same study (ie, substance use). ^b^Past-year posttraumatic stress disorder (PTSD) prevalence rates for contextual comparison are from Kang et al^26^ for postdeployment Gulf War veterans from 1995 to 1997, the National Epidemiologic Survey on Alcohol and Related Conditions-III for the United States from 2012 to 2013,^27^ and the World Health Organization World Mental Health Surveys for the global average from 2001 to 2012.^28^ Lifetime (>1 year) PTSD prevalence rates are from the National Vietnam Veterans Readjustment Study for male Vietnam-era veterans from 1986 to 1988,^29^ the National Epidemiologic Survey on Alcohol and Related Conditions-III for the United States,^27^ and the World Mental Health Surveys for the global average.^28^ Lifetime PTSD prevalence rates are higher among Vietnam veterans than those of more recent deployments.^30^

At the time of survey (August 2019 to April 2020), presumptive mental disorders were highly prevalent among Flint residents (Table 2) and enhanced in comparison with prior rates reported among Michigan residents as a whole, military veterans after deployment, the US population, and estimated global averages (Figure 2).^24,25,26,27,28,29,30^ Of the Flint residents surveyed, 435 (22.1%; 95% CI, 20.1%-24.2%) met *DSM-5* criteria for past-year depression, 480 (24.4%; 95% CI, 22.2%-26.6%) for past-year PTSD, and 276 (14.0%; 95% CI, 12.3%-15.7%) for both disorders.

**Table 2.** Weighted Prevalence of and Factors Associated With Presumptive Depression, PTSD, and Comorbidity Among Flint Residents 5 Years After the Onset of the Water Crisis

Factor	RR (95% CI)		
	Depression	PTSD	Comorbidity
Prevalence, No. (%)	435 (22.1)	480 (24.4)	276 (14.0)
Sociodemographic characteristics			
Race			
Black	0.83 (0.68-1.01)	0.94 (0.78-1.13)	0.81 (0.63-1.05)
>1 Race	1.52 (1.05-2.21)^a^	1.31 (0.89-1.94)	1.90 (1.21-2.97)^a^
Other^b^	1.07 (0.67-1.70)	0.86 (0.51-1.45)	1.20 (0.68-2.12)
White	1 [Reference]	1 [Reference]	1 [Reference]
Sex			
Female	1 [Reference]	1 [Reference]	1 [Reference]
Male	0.72 (0.59-0.89)^c^	0.80 (0.65-0.97)^a^	0.69 (0.52-0.91)^c^
Income, per year, $			
≥25 000	1 [Reference]	1 [Reference]	1 [Reference]
<25 000^c^	1.39 (1.14-1.69)	1.65 (1.36-2.01)	1.72 (1.32-2.25)
Water crisis–related factors^d^			
Believe that health was harmed by exposures^c^^,^^e^	2.23 (1.80-2.76)	1.66 (1.36-2.03)	2.06 (1.56-2.71)
Have low confidence in official information^c^^,^^f^	1.47 (1.17-1.83)	1.44 (1.16-1.78)	1.50 (1.12-2.02)
Non–water-crisis–related factors^d^			
Past exposure to any potentially traumatic events^c^^,^^g^	2.73 (2.00-3.74)	4.55 (3.02-6.86)	5.06 (2.99-8.58)
Exposure to physical or sexual assault/abuse^c^	3.41 (2.47-4.70)	6.28 (4.15-9.50)	7.30 (4.30-12.42)
Exposure to a non-assault traumatic event^c^	1.94 (1.37-2.75)	2.53 (1.62-3.95)	2.41 (1.34-4.33)
Low social support^c^^,^^h^	2.42 (1.84-3.18)	2.58 (1.94-3.43)	2.74 (1.92-3.91)

Abbreviations: PTSD, posttraumatic stress disorder; RR, risk ratio.

^a^ Significant associations at α = .05.

^b^ Other race category includes respondents identifying as Asian, Native American, Native Hawaiian or Pacific Islander, or other.

^c^ Significant association at α adjusted for the number of tests within the domain (eg, race, sex, income, etc) using the Bonferroni correction.

^d^ Associations adjusted for race, sex, and income.

^e^ Respondents who felt that their health or the health of a family member was moderately or greatly harmed by exposure (1003 of 1970 [50.9%]).

^f^ Respondents who reported little to no confidence in public official–provided information on water safety, at the start of the crisis and 5 years later (1294 of 1970 [65.7%]).

^g^ Respondents previously exposed to a potentially traumatic event (1480 of 1970 [75.1%]) compared with those with no past exposure. Of those with previous exposure, 771 (52.1%) reported exposure to physical or sexual assault or abuse, and 709 (47.9%) reported exposure only to another form of potentially traumatic event (eg, a serious illness, a natural disaster). Further details appear in eAppendix 1 in the Supplement.

^h^ Respondents with a score of 15 or less on a scale (range, 5-20) assessing social support (1223 of 1785 [68.5%]).

Secondary follow-up tests investigated 3 categories of factors potentially associated with mental disorder (Table 2): (1) sociodemographic characteristics (race and ethnicity, sex, and income); (2) psychological risk factors related to the water crisis (concerns about health problems following contaminated-water exposure and low confidence in public-official information); and (3) psychological risk factors unrelated to the water crisis (past PTE exposure and low social support). Given the large number of statistical tests, Table 2 highlights associations that were statistically significant at α = .05 and after adjusting the α for multiple comparisons within a domain using the Bonferroni correction.

Sociodemographic factors were significantly associated with presumptive mental disorder 5 years after onset of the water crisis. Specifically, after adjustment for multiple comparisons, race was not significantly associated with mental health disorders, but sex and income were. Men were 28% less likely than women to meet criteria for depression (risk ratio [RR], 0.72; 95% CI, 0.59-0.89) and 20% less likely to meet criteria for PTSD, although this latter association was only significant at α = .05 (RR, 0.80; 95% CI, 0.65-0.97). Compared with more affluent participants, those reporting income below $25 000 per year were significantly more likely to experience depression, PTSD, and comorbid disorder.

Thoughts and beliefs about the water crisis were also significantly associated with risk for presumptive mental disorder. After adjustment for sociodemographic covariates and multiple comparisons, respondents who felt that the water crisis had affected their health or their family’s health were significantly more likely than their peers to meet criteria for depression (RR, 2.23; 95% CI, 1.80-2.76), PTSD (RR, 1.66; 95% CI, 1.36-2.03), or comorbid disorder (RR, 2.06; 95% CI, 1.56-2.71). Likewise, respondents who consistently voiced low confidence in the accuracy of public-official information about the water crisis were significantly more likely than their peers to meet criteria for depression (RR, 1.47; 95% CI, 1.17-1.83), PTSD (RR, 1.44; 95% CI, 1.16-1.78), and comorbid disorder (RR, 1.50; 95% CI, 1.12-2.02).

Preexisting risk factors for poor mental health were also significantly associated with risk for presumptive mental disorders. Individuals with past exposure to a PTE, such as a life-threatening illness, serious accident, or previous disaster, were significantly more likely than their peers to experience depression (RR, 2.73; 95% CI, 2.00-3.74) or PTSD (RR, 4.55; 95% CI, 3.02-6.86) and were 5 times more likely to experience comorbid disorder (RR, 5.06; 95% CI, 2.99-8.58). Separating PTE exposures into those specifically related to physical or sexual assault vs all others indicated that, as has been previously shown,^31^ individuals with assault-related PTEs were significantly more likely to have mental disorder than individuals exposed only to non-assault PTEs (eg, comorbid disorder: RR, 7.30 [95% CI, 4.30-12.42] vs RR, 2.41 [95% CI, 1.34-4.33]). Likewise, individuals with low social support, meaning few to no people to confide in, relax with, or ask for advice, were significantly more likely than their peers to experience depression (RR, 2.42; 95% CI, 1.84-3.14) or PTSD (RR, 2.58; 95% CI, 1.94-3.43) and were nearly 3 times more likely to experience comorbid disorder (RR, 2.74; 95% CI, 1.92, 3.91).

eTable 3 in the Supplement presents conservative sensitivity tests regressing all potential factors associated with mental health disorders onto the mental health outcomes simultaneously. eTable 4 in the Supplement presents the intercorrelation of the risk factors and outcomes.

### Mental Health Service Use

Only 685 respondents (34.8%) reported ever being offered mental health services to assist with concerns or problems associated with the water crisis. Somewhat unexpectedly, Black residents were more likely to be offered these services than White residents (RR, 1.21; 95% CI, 1.04-1.40), as were those making less than $25 000 a year compared with more affluent residents (RR, 1.19; 95% CI, 1.01-1.40). Women were somewhat less likely to be offered services than men (RR, 0.87; 95% CI, 0.75-1.00), although this result was not statistically significant.

Although a minority of Flint residents were offered mental health services, most of those offered services reported using them (543 [79.3%]). After adjustment for race, income, and sex, individuals who utilized mental health services were 36% less likely to have presumptive depression (adjusted RR, 0.64; 95% CI, 0.50-0.83) and 10% less likely to have presumptive PTSD (adjusted RR, 0.90; 95% CI, 0.72-1.13), although the latter result was not statistically significant.

## Discussion

This assessment of a household probability sample of adult Flint residents 5 years after the onset of the water crisis yielded 4 principal findings. First, expanding on previous findings of problems identified during^11,12,13,32^ and just after the crisis,^14,15^ we found that mental health concerns remained significantly elevated in Flint relative to other populations. Overall, 1 in 5 surveyed Flint residents met criteria over the past year for presumptive major depression, 1 in 4 for presumptive PTSD, and 1 in 10 for comorbid depression and PTSD. Applying these weighted sample prevalence estimates to the population suggests that approximately 22 600 Flint residents may have had depression, 25 000 may have had PTSD, and 14 300 may have had comorbid depression and PTSD in 2019 to 2020.

Although estimated rates of past-year mental disorder vary by population, these presumptive prevalence estimates exceed regional, national, and global benchmarks. The prevalence of presumptive past-year depression in Flint (22.1%) is 2-fold greater than Michigan (9.4%), US (7.8%), and global (7.2%) estimated base-rates,^24,25^ and the prevalence of presumptive past-year PTSD is 2- to 5-fold greater than base-rates among veterans after deployment (12.1%), the general US population (4.7%), and estimated global averages (2.8%).^27,28,29^ (In 2019-2020, 93.4% of veterans reported at least 1 exposure to a PTE.)^30^ Our estimates fall within the range of those previously reported for Flint residents using brief screening surveys throughout and just after the water crisis. In 2016, 29.6% of Flint residents were estimated to meet screening criteria for current depression (past 2 weeks),^13,15^ a number that decreased to 13.1% by 2018.^15^ In 2015 to 2016, 20.4% of Flint residents were estimated to meet screening criteria for current PTSD (past month),^11^ a number that increased to 29.0% by 2019.^14^ Our estimates are also lower than those reported among other communities exposed to neurotoxicants through human-caused technological disasters, including residents of a town exposed to a severe chlorine gas disaster (36.9% screened positive for PTSD within 1 year of the disaster)^33^ and of a town exposed to widespread methyl parathion contamination (55.0% screened positive for depression within 1 year of disaster identification).^34^

Second, a Flint resident’s risk of having presumptive depression or PTSD half a decade after the water crisis began was significantly associated with their experiences, thoughts, and beliefs concerning the water crisis. Individuals who believed that their or their family’s health was moderately or greatly harmed by the water crisis were 123% more likely than their peers to have depression, 66% more likely to have PTSD, and 106% more likely to have comorbid depression and PTSD. Likewise, individuals who reported low confidence in the information about water safety provided by authorities were significantly more likely to experience a diagnosable mental disorder 5 years into the crisis. Notably, longitudinal surveys suggest that Flint residents’ thoughts and beliefs about the water crisis have changed since the crisis onset (eg, each year of the crisis, fewer residents reported feeling “overlooked by decisionmakers” or concerned that the crisis “compromised their health”); this may reflect a productive avenue for further reducing mental disorder in the community.^15^

Third, in this predominantly low-income, African American community, demographic and psychological risk factors unrelated to the water crisis were significantly associated with mental disorder 5 years later. Individuals reporting the lowest income (<$25 000), lack of social support, and previous exposure to PTEs were significantly more likely to experience presumptive depression, PTSD, and comorbid mental disorder. Notably, disorder prevalences were 3- to 5-fold greater among Flint residents with prior exposure to PTEs, and 3- to 7-fold greater among residents exposed to physical or sexual assault. This has implications for future research and public-health responses following disasters. For researchers, it highlights the need for postdisaster surveys to assess exposure to PTEs involving physical and sexual assault in addition to the index disaster exposure. For policy makers, it suggests that incorporating brief screening measures for this type of PTE and for social support could lead to better identification of individuals most in need of mental health services after disasters. Lastly, these findings suggest that the water crisis could have exacerbated existing mental health disparities in Flint—a possibility that should be investigated in future studies.

Fourth, while there have been efforts after the water crisis was discovered to restore water quality, remove lead service lines, improve child development outcomes, and hold public officials to account, our results suggest that it is likely that the provision of mental health services has not yet matched community need. Just under three-quarters of adults in our sample reported never having been offered mental health services to address their emotional and behavioral concerns related to the water crisis, despite consistent evidence of widespread community need early and throughout the crisis.^11,12,13,32^

Collectively, these results suggest that (1) there is a large, unmet mental health need in the Flint community 5 years after the onset of the water crisis and (2) this need for mental health services is unlikely to remit without a comprehensive, systematic, and coordinated response from the local, state, and federal governments and public health communities. Encouragingly, the vast majority of Flint residents (79.3%) who were offered mental health services after the onset of the crisis utilized them, and those who did so were significantly less likely to meet criteria for presumptive depression. Unfortunately, they were not necessarily less likely to meet criteria for PTSD, a phenomenon that may reflect low access to PTSD-specific treatments or the possibility that the water crisis was not identified at the time as a PTE capable of precipitating PTSD.

### Limitations

This study has limitations. First, our prevalence estimates should be viewed as presumptive cases of depression and PTSD because they reflect respondents who met *DSM-5* diagnostic criteria for the disorder as measured by highly structured, well-validated, self-administered interview modules but were not directly confirmed by clinician-administered diagnostic interviews. Structured interview by a mental health professional remains the best practice for diagnosing mental disorders; follow-up studies should confirm our estimates with clinician interviews wherever possible. Second, the overall response rate was only 28.4%, although 89.7% of those who read the consent agreement agreed to participate and completed the survey. While responses were weighted to match Flint demographic characteristics, the final disorder prevalence estimates may not be entirely accurate if there was substantial nonresponse bias unrelated to demographic characteristics. Prevalence estimates could be low if those who responded were less impaired than those who did not or high if those with unmet mental health needs were more motivated to respond. Notably, the modest response rate is consistent with contemporaneous large-scale community surveys, which have demonstrated consistently declining response rates over the past 30 years.^35^ In light of this widespread time-of-survey phenomenon, response weighting is considered an effective way to mitigate potential nonresponse bias in outcome distributions due to lower response rates.^36^ Third, the study was not longitudinal, so we cannot establish temporal or causal relations among study variables. Fourth, we did not measure actual lead exposure, just perception thereof; future research should include biomarkers of exposure.

## Conclusions

Five years after the onset of the Flint water crisis, high rates of presumptive depression, PTSD, and comorbid mental disorder were found among adults living in the Flint community, although it is important to note that many adults were resilient. These disorders were associated with aspects of the water crisis as well as with preexisting risk factors, including previous exposure to PTEs, particularly assault-related PTEs. These findings suggest that community-level public works environmental disasters have large-scale and lasting psychological sequelae. The Flint community may require more concerted mental health resources to meet continued psychiatric need.

## Notes

Supplement. **eAppendix 1.** Additional Details on the Assessment of Prior Exposure to Potentially Traumatic Events, PTSD, and Social Support **eTable 1.** Prevalence of Prior Exposure to PTEs **eTable 2.** Prevalence of “None of the Time” Responses to Items Assessing Social Support **eAppendix 2.** Additional Details on Sampling Strategy, Data Collection Method, and Weighting **eTable 3.** Simultaneous Adjustment of All Factors Associated With Depression, PTSD, and Comorbidity **eTable 4.** Intercorrelation of the Primary Study Variables
eReferences.
