# Error in Discussion

**DOI:** 10.1001/jamanetworkopen.2022.40010

**Published:** 2022-10-11

In the Original Investigation titled “Prevalence of Depression and Posttraumatic Stress Disorder in Flint, Michigan, 5 Years After the Onset of the Water Crisis,”^1^ published September 20, 2022, there was an error in the Discussion section. In the first paragraph, the number of Flint residents who may have had depression, posttraumatic stress disorder (PTSD), and comorbid depression and PTSD should have read 13 600, 15 000, and 8600, respectively. This article has been corrected.^1^
