# Supplementary material for: Prevalence of Depression and Posttraumatic Stress Disorder in Flint, Michigan, 5 Years After the Onset of the Water Crisis
Source: JAMA Netw Open. 2022 Sep 20;5(9):e2232556. doi: 10.1001/jamanetworkopen.2022.32556 (PMC9490512; doi:10.1001/jamanetworkopen.2022.32556)
Supplement: Supplement. — eAppendix 1. Additional Details on the Assessment of Prior Exposure to Potentially Traumatic Events, PTSD, and Social Support eTable 1. Prevalence of Prior Exposure to PTEs eTable 2. Prevalence of “None of the Time” Responses to Items Assessing Social Support eAppendix 2. Additional Details on Sampling Strategy, Data Collection Method, and Weighting eTable 3. Simultaneous Adjustment of All Factors Associated With Depression, PTSD, and Comorbidity eTable 4. Intercorrelation of the Primary Study Variables eReferences. [file jamanetwopen-e2232556-s001.pdf]

Supplemental Online Content

Reuben A, Moreland A, Abdalla SM, et al. Prevalence of depression and posttraumatic stress disorder in Flint, Michigan, 5 years after the onset of the water crisis. *JAMA Netw Open.* 2022;5(9):e2232556. doi:10.1001/jamanetworkopen.2022.32556

eAppendix 1. Additional Details on the Assessment of Prior Exposure to Potentially Traumatic Events, PTSD, and Social Support

eTable 1. Prevalence of Prior Exposure to PTEs

eTable 2. Prevalence of “None of the Time” Responses to Items Assessing Social Support

eAppendix 2. Additional Details on Sampling Strategy, Data Collection Method, and Weighting

eTable 3. Simultaneous Adjustment of All Factors Associated With Depression, PTSD, and Comorbidity

eTable 4. Intercorrelation of the Primary Study Variables

eReferences.

This supplemental material has been provided by the authors to give readers additional information about their work.

© 2022 Reuben A et al. *JAMA Network Open.*

eAppendix 1. Additional Details on the Assessment of Prior Exposure to Potentially Traumatic Events, PTSD, and Social Support

Potentially Traumatic Events

Prior exposure to 11 potentially traumatic events (PTEs)* was assessed using items from the National Stressful Events Survey (NSES) PTSD Module developed in conjunction with the APA DSM-5 PTSD Workgroup [1]. Respondents were told, "Some people who have been through stressful experiences like the Flint Water Crisis have also had other events or experiences that may have been extraordinarily stressful or disturbing. During your lifetime, including when you were a child, have any of the following things happened to you?" e**Table 1**, below, presents the individual items assessing PTE exposure and the prevalence of "yes" answers in the sample.

Assessment of PTSD

PTSD was measured using the National Stressful Events Survey (NSES) PTSD Module developed in conjunction with the APA DSM-5 PTSD Workgroup.[1] The NSES PTSD measure is designed to mimic a highly structured clinical interview that strictly follows DSM-5 PTSD diagnostic criteria including that the symptoms produce either substantial distress or impairment in role functioning. Instead of being administered by an interviewer, it is self-administered by the respondent either in web-based form or via a hard-copy paper that is completed and mailed back. It uses branching format programing in both the web and paper hard-copy versions.

The first step in PTSD assessment is assessment of PTE exposure as described above. The purpose of this is to prime the respondent to think about exposure to these PTEs in reference to the PTSD symptoms they will next be asked about. To provide a better idea about the content and format of the module, a copy of instructions to respondents, a sample of eight of the 20

© 2022 Reuben A et al. *JAMA Network Open.*

PTSD symptoms (two each from PTSD symptom criteria B, C, D, and E), and two questions measuring PTSD symptom-related distress/functional impairment are presented below.

Instructions

Many people develop problems or difficulties after extremely stressful events/experiences such as the ones we just asked you about or the Flint Water crisis. Please tell us if you have EVER had any of the following problems, including if you have the problem currently.

36. You had repeated, distressing memories about a stressful experience from the past that just popped into your head when you weren't expecting it or when something reminded you of the stressful experience?
1 Yes **[CONTINUE]**
2 No **[SKIP TO Q37]**
8 Don't know/Not sure **[SKIP TO Q37]**
9 I prefer not to answer **[SKIP TO Q37]]**

36A. When is the last time one of these distressing memories popped into your head?
1 Within the past month **[CONTINUE]**
2 Within the past 6 months **[SKIP TO Q37]**
3 Within the past year **[SKIP TO Q37]**
4 More than 1 year ago **[SKIP TO Q37]**
8 Don't know/Not sure **[SKIP TO Q37]**
9 I prefer not to answer

36B. How much have you been bothered by these distressing memories during the past month?
1 Not at all
2 A little bit
3 Moderately
4 Quite a bit
5 Extremely
8 Don't know/Not sure
9 I prefer not to answer

38. You had "flashbacks," that is, you suddenly acted or felt as if a stressful experience from the past was happening all over again?
1 Yes **[CONTINUE]**
2 No **[SKIP TO Q39]**
8 Don't know/Not sure **[SKIP TO Q39]**
9 I prefer not to answer

[**Same follow up questions as above**]

41. You tried to avoid having thoughts, feelings, or physical sensations that reminded you of a stressful event/experience that happened to you?
1 Yes **[CONTINUE]**
2 No **[SKIP TO Q42]**
8 Don't know/Not sure **[SKIP TO Q42]**
9 I prefer not to answer **[SKIP TO Q42]**

[Same follow up questions as above]

42. You went out of your way to avoid people, places, activities, conversations, objects, or situations that reminded you of a stressful experience?

© 2022 Reuben A et al. *JAMA Network Open.*

1 Yes **[CONTINUE]**
2 No **[SKIP TO Q43]**
8 Don't know/Not sure **[SKIP TO Q43]**
9 I prefer not to answer **[SKIP TO Q43]**
42A.

[**Same follow up questions as above]**

43. Sometimes people are not able to remember an important aspect of a stressful experience. Did this ever happen to you after a stressful experience?
1 Yes **[CONTINUE]**
2 No **[SKIP TO Q44]**
8 Don't know/Not sure **[SKIP TO Q44]**
9 I prefer not to answer **[SKIP TO Q44**

[Same follow up questions as above]

45.You changed the way you thought about yourself or about the future after a stressful event/experience? That is, you thought that you were a really bad person, that you could never trust anyone again, that nothing good could ever happen, that you would never be able to have a career, a good relationship, marriage, or children, or that your life would somehow be cut short?
1 Yes **[CONTINUE]**
2 No **[SKIP TO Q45]**
8 Don't know/Not sure **[SKIP TO Q45]**
9 I prefer not to answer **[SKIP TO Q45]**

[Same follow up questions as above]

50. You had a period of time lasting more than a few days when you were extremely irritable or angry to the point that you yelled at other people, got into fights, or destroyed things?
1 Yes **[CONTINUE]**
2 No **[SKIP TO Q51]**
8 Don't know/Not sure **[SKIP TO Q51]**
9 I prefer not to answer **[SKIP TO Q51]**

[Same follow up questions as above]

53. You felt jumpy or easily startled when you heard an unexpected noise?
1 Yes **[CONTINUE]**
2 No **[SKIP TO Q54]**
8 Don't know/Not sure **[SKIP TO Q54]**
9 I prefer not to answer **[SKIP TO Q54]**

[Same follow up questions as above]

PTSD Criterion G: "The disturbance causes clinically significant distress or impairment in social, occupational, or other important areas of functioning."
56. When you experienced these problems that we have just asked you about, how distressing was it for you?
1 Not at all
2 A little bit
3 Moderately
4 Quite a bit
5 Extremely
8 Don't know/Not sure
9 I prefer not to answer

© 2022 Reuben A et al. *JAMA Network Open.*

Bad moods, bad feelings, and bad memories like those we asked you about can sometimes affect your life in other ways.

57. When you had these bad moods, feelings, and memories, did they ever affect your work or school performance, your relations with your family or friends, or your ability to take care of things in your personal life?
1 Yes
2 No
8 Don't know/Not sure
9 I prefer not to answer

Validity and reliability of the PTSD assessment module

The modified NSES PTSD Module is not a "gold standard" clinician administered semi-structured interview such as the Structured Clinical Interview for DSM-5 PTSD Module (SCID-5)[3] or the Clinician-Administered PTSD Scale for DSM-5 (CAPS-5).[4] This raises the question of whether it is sufficiently reliable and valid to produce acceptably accurate estimates of presumptive PTSD in epidemiological studies of communities that have experienced natural or manmade disasters. To address this question, we provide the following information. First, the past month internal consistency reliability for the 20 PTSD symptoms was .93, which is classified as excellent. Second, data from the DSM-IV PTSD Field Trial using the DSM-IV form of our module found good agreement between PTSD diagnoses obtained by lay interviewers using our module and PTSD diagnoses by mental health professionals using the semi-structured SCID DSM-IV PTSD Module (kappa = .77), confirming that our measure is reliable when compared to a "gold standard" clinician interview. Finally, the NSES PTSD Module was used in a DSM-5 PTSD work group commissioned study[1] and produced a national U.S. past-year DSM-5 PTSD prevalence estimate of 4.7%. A large national study that assessed PTSD using DSM-5 criteria with face-to-face in-person highly structured interviews replicated our findings exactly (i.e., the past year prevalence was 4.7%).[5] Both estimates were cited in the APA's DSM-5-TR Manual as national prevalence estimates for PTSD. These data suggest that the NSES PTSD

© 2022 Reuben A et al. *JAMA Network Open.*

Module has sufficient reliability, validity, and acceptance in the PTSD field to produce justifiable estimates of presumptive PTSD in post-disaster studies including this one in Flint.

<u>Social Support</u>

Social support was measured via a modified five-item version of the Medical Outcomes Study module that assesses social support over the past six months. Respondents were asked how often they had someone available to "help you if you were confined to bed", "give good advice about a crisis", "get together with for relaxation, "confide in or talk with about your problems", and "love you and make you feel wanted". Response options ranged from "none of the time" (score=1) to "all of the time" (score=4) (scale range 5-20). This scale has good reliability (α=0.86). **eTable 2**, below, presents the prevalence of "none of the time" responses in the sample. Based on results from prior studies of the 9/11 terrorist attacks [6] and hurricanes in Florida [7], we defined low social support as a score of ≤15. 70.7% of respondents reported low social support.

© 2022 Reuben A et al. *JAMA Network Open.*

eTable 1. Prevalence of Prior Exposure to PTEs

PTE item: "Did you ever…"	Unweighted N	Unweighted prevalence (%)	Weighted prevalence (%)
1. Serve in a war zone, or serve in a noncombat job that exposed you to war-related casualties (for example, as a medic or on graves registration duty)?	68	3.5	3.7
2. Have a serious car accident or some other type of serious accident?	574	29.3	29.1
3. Experience a major natural or technological disaster, such as a fire, tornado, hurricane, flood, earthquake, or chemical spill?	346	17.7	16.9
4. Have a life-threatening illness such as cancer, a heart attack, leukemia, AIDS, or multiple sclerosis?	319	16.3	15.3
5. Before age 18, were you ever physically punished or beaten by a parent, caretaker, or teacher so that: you were very frightened; or you thought you would be injured; or you received bruises, cuts, welts, lumps, or other injuries?	359	18.3	17.1
**PTE item: "Did you ever…"**	**Unweighted N**	**Unweighted prevalence (%)**	**Weighted prevalence (%)**
6. Not including any punishments or beatings you just reported in the previous question, have you ever been attacked, beaten, or mugged by anyone, including friends, family members or strangers?	600	30.6	29.4
7. Has anyone ever used physical force or threats of force to make you have some type of unwanted sexual contact? Note: By sexual contact, we mean any contact between someone else and your private sexual parts or between you and someone else's private sexual parts.	362	18.4	15.3
8. Has someone ever had unwanted sexual contact with you when you were passed out or unable to consent or stop what was happening due to the effects of alcohol or drugs? This includes unwanted touching of private sexual parts or unwanted sexual intercourse, oral sex, or anal sex.	190	9.7	8.3

© 2022 Reuben A et al. *JAMA Network Open.*

9. Have you been in any other situation in which you were seriously injured or in which you feared you might be seriously injured or killed?	485	24.8	23.8
10. Have you had a close family member or friend die violently, for example, in a serious car crash, suicide, murder, shooting, or terrorist attack?	920	46.9	46.8
11. Have you witnessed a situation in which someone was seriously injured or killed, or have you ever witnessed a situation in which you thought someone would be seriously injured or killed?	705	35.9	36.4
**Summary Scores**	**Unweighted N**	**Unweighted prevalence (%)**	**Weighted prevalence (%)**
Any exposure to a potentially traumatic event (items 1-11)	1,512	76.8	75.1
Any exposure to physical or sexual assault (items 5-8)	816	41.4	39.2

[*] According to the DSM-5, the Criterion A definition of a PTE is: "Exposure to actual or threatened death, serious injury, or sexual violence in one (or more) of the following ways: 1) directly experiencing the traumatic event(s); 2) witnessing, in person, the event(s) as it occurred to others; 3) learning that the traumatic event(s) occurred to a close family member or close friend; or 4) is experiencing repeated or extreme exposure to aversive details of the traumatic event(s)." Examples of qualifying PTEs are described on pages 274-275 of the DSM-5 manual.[2]

© 2022 Reuben A et al. *JAMA Network Open.*

eTable 2. Prevalence of "None of the Time" Responses to Items Assessing Social Support

**Social support item: "Do you have someone available to…"**	**Unweighted N for "none of the time" responses**	**Unweighted prevalence (%)**	**Weighted prevalence (%)**
Help you if you were confined to bed?	666	34.2	33.2
Give good advice about a crisis?	488	25.0	23.8
Get together with for relaxation?	455	23.4	23.1
Confide in or talk about your problems?	351	18.0	18.2
Love you and make you feel wanted?	275	14.1	14.2

© 2022 Reuben A et al. *JAMA Network Open.*

eAppendix 2. Additional Details on Sampling Strategy, Data Collection Method, and Weighting

Sampling Strategy and Data Collection Method

The goal of our sampling strategy was to locate a household probability sample of adults (age 18 and older) who had potential exposure to contaminated water during the Flint Water Crisis. Potential exposure to contaminated water in Flint was sufficiently widespread that it was necessary to include all Flint households in the sampling frame. The three major household probability sampling options we considered were: 1) in person sampling of households; 2) dual-frame (i.e., landline and mobile telephone) random digit dialing (RDD) sampling in which phone numbers are called randomly to identify individuals who reside in households within the geographic area; and 3) address-based sampling (ABS) in which letters describing the study are mailed to randomly selected households within the specified geographic area. Each method has advantages and disadvantages.

In person sampling surveys were once considered to be state of the art and were widely used. However, they are very expensive and cost 3-4 times as much as RDD dual-frame or ABS surveys. When 98% of US households had landlines, RDD sampling became a widely-used, viable option. However, the proportion of households with landline phones has dropped dramatically over the past twenty years, necessitating use of RDD dual-frame sampling including mobile phones. RDD dual-frame sampling surveys have much lower response rates than previous landline-only RDD surveys. Consequently, ABS sampling surveys, which are comparable in cost to RDD dual-frame surveys but much less expensive than in-person sampling surveys, are now considered to be a first-line option for household surveys, particularly when there is a need to sample households in a specified geographic area.

Given budgetary constraints and the need to limit the sampling frame to households in Flint, we elected to use ABS sampling. The target population for the project was adults age 18 and over who lived in Flint, Michigan for at least some time after the onset of the water crisis (April 2014). The sample design included an address-based sample of addresses in Flint, Michigan, drawn from the US Postal Service's Delivery Sequence File (DSF). The address-based sample was provided by Marketing Systems Group and consisted of a random sample of 10,000 Flint addresses.

Five basic methods are used to collect data in household surveys: 1)face-to-face in person interviews; 2) face-to-face interviews using computer-administered modules for sensitive content items (e.g., respondents are given a tablet with earphones by the interviewer in which there is computerized administration of the survey; instructions and questions are recorded and read out loud to respondents who then key in their answers); 3) telephone interviews: 4) paper surveys that are mailed to respondents; and 5) web-based, self-administered surveys that are completed by respondents online. The first three of these methods are primarily used in the context of in-person or telephone surveys, but ABS surveys typically use one or both of the paper survey and web-based survey methods. The most commonly-used method in ABS surveys is push-to-web with a follow-up in which paper surveys are mailed to those who do not respond to requests to complete the web-based survey. We decided to use this push-to-web with mailed paper survey follow-up data collection method in the Flint survey.

Another justification for using these two data collection modes is that the Flint survey contains many questions on sensitive topics (e.g., potentially traumatic event exposure, alcohol and drug use, mental health problems), and there is considerable research showing that surveys collecting data using live interviewers often obtain lower and less accurate reports of experiences

© 2022 Reuben A et al. *JAMA Network Open.*

that are sensitive, embarrassing, or stigmatizing than when the same survey does not include a face-to-face or telephone interviewer.[e.g.,8–10] Neither web or paper surveys require contact with a live interviewer, and studies comparing results using paper vs web surveys have not found meaningful differences using these two modes. [e.g.,11,12]

All addresses were sent an invitation to the web survey via postal mail. Participants in this "push-to-web" portion received a packet in the mail with information about the project, a URL directing them to the online survey, and a unique project ID number. All addresses were also sent a second mailing which consisted of a postcard thanking those that have completed and reminding those that have not. The last mailing included a paper copy of the needs assessment and a business reply envelope (BRE). The mailing was sent to address based participants who had not responded to the initial invitation to complete the online needs assessment and also excluded any undeliverable addresses from previous mailings.

Weighting

Two final weights were created for the 1,970 respondents from the address-based sample. The final weight consists of an adjustment for household size, assessment non-response, and a final calibration of the characteristics of respondents to the adult population of Flint, Michigan. Prior to weighting, missing data in the variables used in the weighting were imputed using the modal response in each assessment mode. Respondent's age was not included in the calibration weighting adjustment due to the high rates of missing data.

A weighting adjustment was made to reflect the fact that adults living in households with more people had a lower probability of being selected to complete the needs assessment. The adjustment for household size (BSW_HH) is equal to the size of the household, capped at a value of two household members to prevent undue variance in the weights.

A total of 10,000 addresses were sampled for the Flint survey. To the extent that those responding to the survey may be different from those who did not, there is a risk that estimates could be subject to nonresponse bias. Nonresponse weights based on propensity score adjustments were computed to adjust for differential nonresponse to the survey. The sample frame provided by Market Systems Group includes variables appended to each address that describe the housing unit as well as demographic characteristics of the occupant(s). These variables were used to predict the likelihood that a household associated with the address would respond to the survey.

A logistic regression model was estimated in which responding to the survey was regressed on the following flags provided by MSG based on proprietary marketing data (we cannot treat them as true values, but they are helpful in creating response propensity models): 1); Zip Code; 2) Dwelling type (single vs. multi-family); 3) Tenure of the housing unit (rented vs. owned); 4) Presence of a landline telephone in the housing unit; 5) Presence of a child in the household; 6) Number of adults in the household; 7) Sex; 8) Education level; 9) Race (Black/African American or not); 10) Marital status; and 11) Income.

The response propensity model was estimated using all sampled addresses, excluding cases known to be ineligible, such as vacant and undeliverable addresses and screen-outs. The estimated propensities were used to divide cases into approximately equal size groups using the quartiles of the estimated propensity score. The nonresponse weight for respondents was computed as the inverse of the response rate in each quartile. This approach helps to protect against model misspecification, relative to using the inverse of the response propensities.

The final baseweight for each respondent (FINAL_BSW) was computed as the product of the household size adjustment and the nonresponse weight. In the final stage of weighting, the

final baseweights for respondents were calibrated to population parameters using raking, or iterative proportional fitting. The characteristics of respondents were aligned to match population benchmarks on sex, education level, race/Hispanic ethnicity, marital status, and household size. The weighting parameters were obtained from an analysis of the Census Bureau's 2018 American Community Survey (ACS) one-year estimates, filtered on aged 18 years and older residing in households in Flint, Michigan. No trimming was conducted on the final raked weights.

.

© 2022 Reuben A et al. *JAMA Network Open.*

eTable 3. Simultaneous Adjustment of All Factors Associated With Depression, PTSD, and Comorbidity

Panel A. Parameter estimates for the association of all potential risk factors with depression, assessed simultaneously.

	RR	95% CI	Standard error	p-value	
Race[a]	1.26	1.05, 1.51	0.12	.012	
Sex[b]	0.76	0.62, 0.92	0.08	.006	
Income[c]	1.16	0.96, 1.40	0.11	.129	
Belief that health was harmed by exposures[d]	1.82	1.47, 2.25	0.20	<.001	
Low confidence in official information[e]	1.11	0.90, 1.37	0.12	.338	
Past exposure to any potentially traumatic events[f]	1.77	1.25, 2.49	0.31	.001	
Exposure to physical or sexual assault/abuse[f]	1.57	1.28, 1.94	.17	<.001	
Low social support[g]	2.07	1.58 2.71	0.28	<.001	

Panel B. Parameter estimates for the association of all potential risk factors with PTSD, assessed simultaneously.

	RR	95% CI	Standard error	p-value	
Race[a]	1.01	.85, 1.21	0.09	.868	
Sex[b]	0.80	0.67, 0.96	0.08	.019	
Income[c]	1.45	1.19, 1.76	0.14	<.001	
Belief that health was harmed by exposures[d]	1.30	1.07, 1.58	0.13	.009	
Low confidence in official information[e]	1.12	0.92, 1.36	0.11	.273	
Past exposure to any potentially traumatic events[f]	2.27	1.45, 3.56	0.52	<.001	
Exposure to physical or sexual assault/abuse[f]	2.34	1.88, 2.92	0.26	<.001	
Low social support[g]	2.11	1.61, 2.78	0.29	<.001	

© 2022 Reuben A et al. *JAMA Network Open.*

Panel C. Parameter estimates for the association of all potential risk factors with comorbid depression and PTSD, assessed simultaneously

	RR	95% CI	Standard error	p-value
Race[a]	1.17	0.92, 1.48	0.14	.194
Sex[b]	0.70	0.54, 0.91	0.09	.008
Income[c]	1.44	1.11, 1.88	0.19	.007
Belief that health was harmed by exposures[d]	1.52	1.16, 2.01	0.22	.002
Low confidence in official information[e]	1.07	0.81, 1.42	0.15	.637
Past exposure to any potentially traumatic events[f]	2.38	1.31, 4.33	0.73	.005
Exposure to physical or sexual assault/abuse[f]	2.75	2.01, 3.76	0.44	<.001
Low social support[g]	2.19	1.55, 3.11	0.39	<.001

Note. CI = Confidence Interval

[a] Coded as Black non-Hispanic (53.6%, reference category) vs. non-Black non-Hispanic

[d] Coded as female (54.5%, reference category) vs male

[d] Coded as ≥ $25,000 per year (56.8%, reference category), versus < $25,000 per year

[d] Respondents who felt that their health or the health of a family member was moderately or greatly harmed by exposure (50.9%).

[e] Respondents who reported little to no confidence in public official-provided information on water safety, at the start of the crisis and 5 years later (65.7%).

[f] Respondents previously exposed to a potentially traumatic event (75.1%) compared to those with no past exposure. Of those with previous exposure, 52.1% reported exposure to physical or sexual assault/abuse and 47.9% reported exposure only to another form of potentially traumatic event (e.g., a serious illness, a natural disaster, etc.).

[g] Respondents with a score ≤15 on a 5-20 range scale assessing social support (70.7%).

eTable 4. Intercorrelation (phi and Pearson's r coefficients) of the Primary Study Variables (mental disorder and risk and resilience factors).

	1. MDD	2. PTSD	3. Co-morbidity	4. Race	5. Sex	6. Income	7. Health harm	8. Low confidence	9. PTE exposure	10. Assault exposure
1. MDD	---									
2. PTSD	.48***	---								
3. Comorbidity	.76***	.72***	---							
4. Race[a]	.05*	.03	.03	---						
5. Sex[b]	-.05*	-.04	-.04	.06*	---					
6. Income[c]	-.07**	-.12***	-.10***	.11***	.03	---				
7. Health harm[d]	.22***	.17***	.16***	-.18***	-.07**	-.14***	---			
8. Low confidence[e]	.10***	.11***	.09***	-.05*	-.07**	-.02	.16***	---		
9. PTE exposure[f]	.16***	.23***	.17***	.08***	.03	-.002	.09***	.09***	---	
10. Assault-PTE exposure[f]	.23***	.35***	.27***	.12***	.02	-.05*	.10***	.10***	.46***	---
10. Low social support[g]	.18***	.19***	.15***	.01	.02	-.16***	.09***	.07**	.07**	.14***

Note. MDD = Major depressive disorder.
*p-values <.05, **p-values <.01, ***p-values <.001
[a] Coded as Black non-Hispanic (53.6%, reference category) vs. non-Black non-Hispanic
[b] Coded as female (54.5%, reference category) vs male
[c] Coded on a 5-point scale of 1(< $25,000 per year) to 5 (≥$100,000 per year)
[d] Respondents who felt that their health or the health of a family member was moderately or greatly harmed by exposure (50.9%).
[e] Respondents who reported little to no confidence in public official-provided information on water safety, at the start of the crisis and 5 years later (65.7%).
[f] Respondents previously exposed to a potentially traumatic event (75.1%) compared to those with no past exposure. Of those with previous exposure, 52.1% reported exposure to physical or sexual assault/abuse and 47.9% reported exposure only to another form of potentially traumatic event (e.g., a serious illness, a natural disaster, etc.).
[g] Respondents with a score ≤15 on a 5-20 range scale assessing social support (70.7%).

eReferences.

1. Kilpatrick DG, Resnick HS, Milanak ME, Miller MW, Keyes KM, Friedman MJ. National estimates of exposure to traumatic events and PTSD prevalence using DSM-IV and DSM-5 criteria. *J Trauma Stress*. 2013;26(5):537-547. doi:10.1002/jts.21848

2. American Psychiatric Association. *Diagnostic and Statistical Manual of Mental Disorders*. 5th ed. American Psychiatric Association; 2013.

3. First MB, Williams JBW, Karg RS, Spitzer RL. *User's Guide for the SCID-5-CV Structured Clinical Interview for DSM-5® Disorders: Clinical Version*. American Psychiatric Publishing, Inc.; 2016:xii, 158.

4. Weathers FW, Bovin MJ, Lee DJ, et al. The Clinician-Administered PTSD Scale for DSM-5 (CAPS-5): Development and initial psychometric evaluation in military veterans. *Psychol Assess*. 2018;30(3):383-395. doi:10.1037/pas0000486

5. Goldstein RB, Smith SM, Chou SP, et al. The epidemiology of DSM-5 posttraumatic stress disorder in the United States: results from the National Epidemiologic Survey on Alcohol and Related Conditions-III. *Soc Psychiatry Psychiatr Epidemiol*. 2016;51(8):1137-1148. doi:10.1007/s00127-016-1208-5

6. Galea S, Ahern J, Resnick H, et al. Psychological sequelae of the September 11 terrorist attacks in New York City. *New England Journal of Medicine*. 2002;346(13):982-987. doi:10.1056/NEJMsa013404

7. Kilpatrick DG, Koenen KC, Ruggiero KJ, et al. The serotonin transporter genotype and social support and moderation of posttraumatic stress disorder and depression in hurricane-exposed adults. *Am J Psychiatry*. 2007;164(11):1693-1699. doi:10.1176/appi.ajp.2007.06122007

8. National Academies of Sciences E. *Measuring Trauma: Workshop Summary*.; 2016. doi:10.17226/23526

9. Tourangeau R, Yan T. Sensitive questions in surveys. *Psychol Bull*. 2007;133(5):859-883. doi:10.1037/0033-2909.133.5.859

10. Parks KA, Pardi AM, Bradizza CM. Collecting data on alcohol use and alcohol-related victimization: a comparison of telephone and Web-based survey methods. *J Stud Alcohol*. 2006;67(2):318-323. doi:10.15288/jsa.2006.67.318

11. Donohue AG, Elliott MN, Batka C, et al. *Sexual Assault and Sexual Harassment in the U.S. Military: Volume 4. Investigations of Potential Bias in Estimates from the 2014 RAND Military Workplace Study*. RAND Corporation; 2016. Accessed July 27, 2022. https://www.rand.org/pubs/research_reports/RR870z6.html

© 2022 Reuben A et al. *JAMA Network Open.*

12. McCabe SE, Diez A, Boyd CJ, Nelson TF, Weitzman ER. Comparing web and mail responses in a mixed mode survey in college alcohol use research. *Addict Behav*. 2006;31(9):1619-1627. doi:10.1016/j.addbeh.2005.12.009

© 2022 Reuben A et al. *JAMA Network Open.*
